# Group C Beta Haemolytic Streptococcus Causing Necrotising Pneumonia Complicated With a Pyopneumothorax

**DOI:** 10.1155/crpu/8885401

**Published:** 2025-10-31

**Authors:** William McLean, Daisy Port, Daniel Sims, Olivia Curtis

**Affiliations:** Royal Surrey County Hospital, Guildford, UK

## Abstract

**Introduction:**

Group C Beta Haemolytic Streptococcus (GCBHS) rarely causes invasive infection but is associated with high mortality. Pneumonia is common; however, severe necrotising pneumonia is very rare, and no previous cases complicated by pyopneumothorax have been described. Associations with male gender and animal exposure have been previously reported in the literature.

**Case:**

A 20-year-old male agriculture student with no past medical history presented with 6 days of productive cough and fever and 2 days of shortness of breath, chest pain, and confusion. He presented with clinical signs of sepsis, developed acute kidney injury (AKI), disseminated intravascular coagulation (DIC), and acute respiratory distress syndrome (ARDS) requiring intubation and ventilation. Initial computed tomography (CT) of the thorax showed right pyopneumothorax, left small pyopneumothorax, bilateral large pulmonary nodules of varying size, some cavitating with progression on serial imaging. Blood cultures grew GCBHS, later showing *Streptococcus dysgalactiae* subsp. *equisimilis* (*SDSE*). Bilateral chest drains were required, with eventual bilateral video-assisted thoracic surgery (VATS) with washout due to nonresolution. After a prolonged intensive care stay and a community course of antibiotics, his follow-up CT of the thorax is improving, and he is planning to return to his studies at agricultural college.

**Conclusion:**

Invasive infection with GCBHS is associated with higher mortality and prolonged hospital admission and may be zoonotic. Of the four reported cases of GCBHS necrotising pneumonia, all were young men with minimal or no past medical history. Half had documented exposure to unwell animals, and half required thoracoscopic intervention.

## 1. Introduction

Invasive infections with Group C Beta Haemolytic Streptococcus (GCBHS) are uncommon, accounting for 0.31% of total bacteraemias [1]. They are associated with the highest mortality rate of Beta Haemolytic Streptococcus (BHS) groups [2, 3] and have shown an association with animal exposure [4] and male sex [5].

Pneumonia was seen in approximately 11% of GCBHS infections [3]; however, necrotising pneumonia has only been reported in three cases [6–8]. Herein, we report the first case of necrotising pneumonia caused by GCBHS complicated by pyopneumothorax in a 20-year-old man with no past medical history, requiring a prolonged hospital admission, bilateral VATS with washout and an extended course of antibiotics.

## 2. Case Presentation

A 20-year-old man presented to the emergency department with a 6-day history of a productive cough, fever, general aches and 2 days of pleuritic chest pain with worsening shortness of breath. A prodrome including a sore throat was noted. He was normally fit and well, BMI 30.3 kg/m^2^, with no previous respiratory, renal or hepatic issues, no history of trauma, no known drug allergies and no regular medications. He was an agriculture student with at least weekly exposure to farm animals including the death of multiple calves and cows on his placement. He drinks alcohol at university, with his weekly intake varying from 0 to 70 units, and reports no history of illicit drug use and no new pets. He travelled to South Africa 6 months prior, including to rural areas. He denies exposure to tuberculosis.

**Figure 1:**
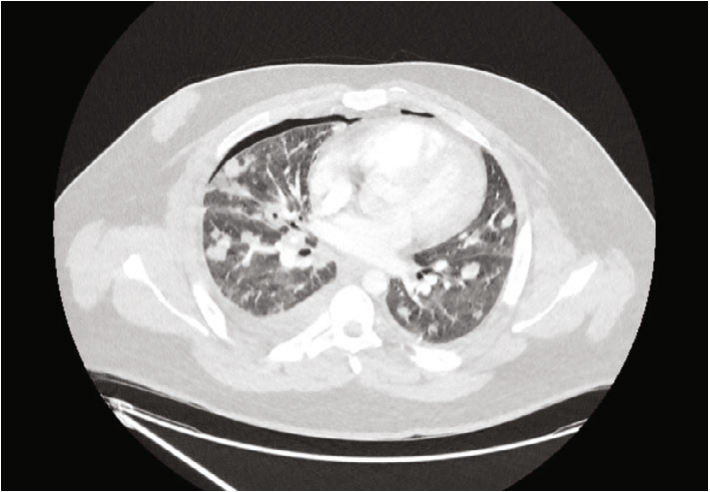
Transverse computed tomography thorax at admission (Day 1) showing right pyopneumothorax, left small pyopneumothorax, bilateral interlobar septal thickening and bilateral pulmonary nodules of varying size, some cavitating.

At presentation in the emergency department, he displayed clinical signs of sepsis: respiratory rate 40–50 respirations/minute, pO2 8.59 kPa on 40% oxygen (oxygen saturation 96%), pulse rate 130 beats/minute, blood pressure 106/76 mmHg, peripherally mottled with prolonged capillary refill time, lactate 3.0 mmol/L with metabolic acidosis, and he was confused (oriented to year, but not month, Glasgow Coma Scale 14/15). Laboratory investigations demonstrated raised inflammatory markers (white cell count 13.3 × 10^9^/L, neutrophils 12.4 × 10^9^/L, CRP 345 mg/L), acute kidney injury (AKI) (urea 18.8 mmol/L, creatinine 224 *μ*mol/L), thrombocytopenia (platelet count 17 × 10^9^/L), mild derangement in coagulation (INR 1.4) and mild liver dysfunction. Computed tomography (CT) pulmonary angiogram showed no pulmonary embolism, a right pyopneumothorax, left small pyopneumothorax, bilateral large pulmonary nodules of varying size, some of which were cavitating (Figure 1).

He rapidly developed acute respiratory distress syndrome (ARDS) necessitating transfer to intensive care for intubation and vasopressor support. A chest drain was inserted on the right and drained frank pus, confirming empyema. Initial antimicrobial treatment with IV ceftriaxone 2 g OD and clarithromycin 500 mg BD was switched to IV ceftriaxone 2 g OD and IV linezolid 600 mg BD with stat gentamicin 400 mg. Intravenous immunoglobulin 195 g was administered when gram-positive cocci were detected on blood culture due to concerns of streptococcal toxic shock syndrome. The pleural pus sample taken on Day 2 of admission grew GCBHS (later identified as *Streptococcus dysgalactiae* subsp. *equisimilis* [*SDSE*]) and another unidentified anaerobe. Ceftriaxone and linezolid were continued to cover GCBHS and bacteria identified on multiplex respiratory polymerase chain reaction (PCR) (BIOFIRE FILMARRAY Pneumonia (PN) Panels, Biomérieux): *Haemophilus influenzae* and *Streptococcus agalactiae*. Due to persistent intermittent pyrexia and progressive elevation of inflammatory biomarkers, ceftriaxone was increased to 2 g BD and linezolid switched to metronidazole 500 mg TDS. Sputum culture for mycobacterium showed *Mycobacterium chimaera* on two samples within 24 h of admission; prolonged culture of five sputum samples and one blood culture were negative.

**Figure 2:**
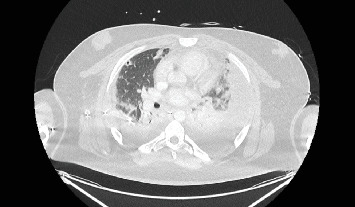
Transverse computed tomography thorax on Day 6 after right intercostal drain insertion on Day 4 demonstrating resolution of pneumothoraces, right intercostal chest drain, bilateral pleural effusions with compressive atelectasis, multifocal consolidation, many demonstrating cavitation.

On Day 6 of admission after minimal clinical improvement, a CT thorax was repeated which showed worsening multifocal consolidation, cavitating opacities throughout the lungs and a new large left pleural effusion (Figure 2), and the patient was transferred to a specialist tertiary centre. A further chest drain was inserted on the left (Day 7), fluid from which was positive for *Fusobacterium necrophorum* DNA by PCR. Anidulafungin was commenced due to raised *β*-d-glucan on Day 7 and discontinued at Day 17 after repeat testing was negative. He received a tracheostomy on Day 16, which was decannulated on Day 26. Bilateral VATS with washout on Day 22 was conducted due to recurrent febrile episodes.

**Figure 3:**
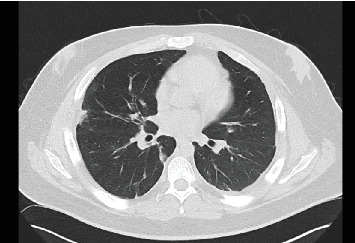
Transverse computed tomography thorax 3 months after presentation demonstrating residual small left pleural effusion, improvement in size of cavitations and resolving consolidation.

The possibility of malignancy was explored and deemed unlikely given imaging findings and presentation. The patient experienced a prolonged hospital admission to rehabilitate following tracheostomy, including a 26-day admission to the intensive care unit, followed by a community prolonged (6 weeks) course of boosted co-amoxiclav (oral co-amoxiclav 625 mg TDS and oral amoxicillin 500 mg TDS). Follow-up CT thorax at 3 months from onset showed improvement, with multiple nodules persisting in both lungs, reducing in size, and with no new nodules or cavitations (Figure 3). He is planning to return to his studies at agricultural college.

## 3. Discussion

The Lancefield classification of GCBHS encompasses several species relevant to human infection. The majority of cases, including the present one, belong to the subspecies *SDSE*, which is also known to express Lancefield Group “G” in addition to Group “C” and consequently some studies combine these two categories [9]. This organism is recognised as a normal constituent of the flora present in the human skin and mucosal linings such as the pharynx and vagina [10]. *SDSE* is a cause of disease in cattle, such as bovine mastitis [11]. Several other species of GCBHS known to cause infections in animals have been recognised to cause infections in humans, namely, *Streptococcus equi* subspecies *zooepidemicus* [4] and *Streptococcus dysgalactiae* subspecies *dysgalactiae* [12, 13]. An early review of 88 GCBHS cases found animal exposure reported in 23.9% of cases [14], and the prevalence of GCBHS has also shown a bias towards males [5]. The patient's close contact with unwell calves and cows as an agriculture student may therefore be relevant and suggest a zoonotic component to his infection with *SDSE*.

A large Canadian study observed that, for 2007–2012, GCBHS accounted for 0.31% of total bacteraemias [1] and 5%–7% of total BHS infections [2, 3, 5]. Despite being the smallest fraction described in these studies, GCBHS demonstrates a higher associated total mortality of 10.3%–22% [2, 3]. Like most BHS, infections with GCBHS most commonly affect the skin and soft tissue [3]; however, they have been well described to affect a multitude of systems, including bone and joint, lower respiratory tract and infective endocarditis [5, 15]. Of these, pneumonia was seen in approximately 11% of GCBHS infections [3]; however, only three previous cases of necrotising pneumonia in relation to GCBHS have been reported.

A severe but rare complication of bacterial pneumonia, necrotising pneumonia, describes parenchymal liquefaction with necrosis leading to the formation of multiple cavitations [16]. Abscess formation with subsequent formation of bronchopulmonary fistulae can lead to pyopneumothoraces if in proximity to the pleura [17]. This was considered the most likely aetiology of the bilateral pyopneumothoraces observed in this case, given the absence of underlying pulmonary pathology or reported history of thoracic trauma.

In addition to GCBHS, several of the organisms isolated during his admission on respiratory PCR (*Haemophilus influenzae* and *Streptococcus agalactiae* initially, *Fusobacterium necrophorum* later in his illness) do have an association with necrotising pneumonia [17, 18]. Given over 90% of lung abscesses contain multiple organisms [19], it is likely these contributed to the patient presentation. However, given that GCBHS was isolated in both the initial blood culture and the purulent pleural fluid sample, this was identified as the most likely primary pathogen.

*Mycobacterium chimaera* was also grown from sputum and identified by whole genome sequencing and is known to very rarely cause invasive lung disease [20]. The majority of these cases are associated with a cardiopulmonary bypass [21, 22] or immunosuppression and note a prolonged latency period [20]. Given the lack of culture positivity, no evidence of immunosuppression and no history of cardiopulmonary bypass, it is unlikely to be relevant to this case.

The three cases of GCBHS necrotising pneumonia previously described do show similarities to our case. All involved young men aged 20, 28 and 40, immunocompetent, with minimal to no past medical history and all survived to discharge. One had a history of potential animal exposure with an unwell dog at home. Management of our case required bilateral VATS with pleural washout; similarly, a previously reported case was managed with bilateral decortication [7]. The remaining two responded to antibiotics with no indication for thoracoscopic intervention or intercostal chest drain [6, 8].

In summary, we present the first reported case of GCBHS (*SDSE*) causing pyopneumothorax and the fourth reported case of GCBHS necrotising pneumonia. There are striking similarities with all four individuals being young men, with 50% reporting exposure to unwell animals. Although often sensitive to common antibiotics, the high morbidity and mortality of invasive GCBHS infections necessitate early treatment and prolonged admission with consideration of instrumentation with intercostal drainage and thoracoscopic procedures.

## 4. Conclusion

• GCBHS is a rare cause of necrotising pneumonia which may be complicated by pyopneumothorax.
• The four cases of GCBHS necrotising pneumonia described in the literature show an association with younger, immunocompetent men, with half documenting exposure to unwell animals.
• GCBHS necrotising pneumonias reported were sensitive to common antibiotics, associated with prolonged admission in an intensive care setting, with consideration of instrumentation with intercostal drainage and thoracoscopic procedures in 50% of described cases.

## Data Availability

Data sharing is not applicable to this article as no datasets were generated or analysed during the current study.
